# Emergence of *Madariaga virus* as a cause of acute febrile illness in children, Haiti, 2015-2016

**DOI:** 10.1371/journal.pntd.0006972

**Published:** 2019-01-10

**Authors:** John A. Lednicky, Sarah K. White, Carla N. Mavian, Maha A. El Badry, Taina Telisma, Marco Salemi, Bernard A. OKech, V. Madsen Beau De Rochars, J. Glenn Morris

**Affiliations:** 1 Emerging Pathogens Institute, University of Florida, Gainesville, FL, United States of America; 2 Department of Environmental and Global Health, College of Public Health and Health Professions, University of Florida, Gainesville, FL, United States of America; 3 Department of Pathology, Immunology, and Laboratory Medicine, College of Medicine, University of Florida, Gainesville, FL, United States of America; 4 Christianville Foundation School Clinic, Gressier, Haiti; 5 Department of Health Services Research, Management, and Policy, College of Public Health and Health Professions, University of Florida, Gainesville, FL, United States of America; 6 Department of Medicine, College of Medicine, University of Florida, Gainesville, FL, United States of America; INDEPENDENT RESEARCHER, UNITED STATES

## Abstract

Madariaga virus (MADV), also known as South American eastern equine encephalitis virus, has been identified in animals and humans in South and Central America, but not previously in Hispaniola or the northern Caribbean. MADV was isolated from virus cultures of plasma from an 8-year-old child in a school cohort in the Gressier/Leogane region of Haiti, who was seen in April, 2015, with acute febrile illness (AFI). The virus was subsequently cultured from an additional seven AFI case patients from this same cohort in February, April, and May 2016. Symptoms most closely resembled those seen with confirmed dengue virus infection. Sequence data were available for four isolates: all were within the same clade, with phylogenetic and molecular clock data suggesting recent introduction of the virus into Haiti from Panama sometime in the period from October 2012-January 2015. Our data document the movement of MADV into Haiti, and raise questions about the potential for further spread in the Caribbean or North America.

## Author summary

Madariaga virus (MADV) is the name given to what used to be called South American eastern equine encephalitis virus (EEEV), based on recent studies suggesting that MADV is distinct genetically from the EEEV circulating in North America. Until now, MADV has been found primarily in animals in South and Central America, with a limited number of human cases reported (most of whom had encephalitis). Our group has been responsible for a series of studies assessing the etiology of acute febrile illness (AFI) among children in a school cohort in Haiti. Unexpectedly, in April, 2015, we identified MADV on viral culture of plasma from a student with AFI in this cohort; an additional seven cases were identified on culture of samples from children with AFI in this same cohort in February, April, and May 2016. On sequence analysis, all strains were very similar genetically, and appear to have come from a strain introduced into Haiti from Panama sometime in the period from October 2012- January 2015. Symptoms of children were similar to those seen with dengue; none had encephalitis. Our data indicate that this virus, which has the potential for causing serious illness, has been recently introduced into Haiti, and raises the possibility that it might move into other parts of the Caribbean or North America.

## Introduction

Madariaga virus (MADV), also known as South American eastern equine encephalitis virus, is an alphavirus in the family *Togaviridae*. Recent ecologic and genetic studies of eastern equine encephalitis virus (EEEV) have demonstrated clear separation between North and South American EEEV strains: North American EEEV cluster in a single genetic lineage (lineage I, in the system proposed by Arrigo *et al*. [1]), with South American EEEV strains, or MADV, clustering in EEEV lineages II, III, and IV. MADV can cause outbreaks in horses, and appears to infect a variety of mammals, including rats and bats, and possibly birds and reptiles [1–3]. However, less than a dozen human cases of MADV infection have been documented, and almost all were encephalitis cases seen as part of an outbreak in Panama in 2010 [3,4]. In population-based serologic surveys in Panama and the Peruvian Amazon, between 2 and 5% of the general population had evidence of prior infection [2,3,5], suggesting that mild or asymptomatic human infection is relatively common. In support of the latter hypothesis, we recently reported isolation of MADV from a child with acute febrile illness (AFI), but no evidence of encephalitis, during the Zika virus (ZIKV) epidemic in Venezuela [6]. The virus has not been previously recognized in Hispaniola or other parts of the northern Caribbean. We report here the apparent recent introduction of MADV into Haiti.

## Methods

**Fig 1:**
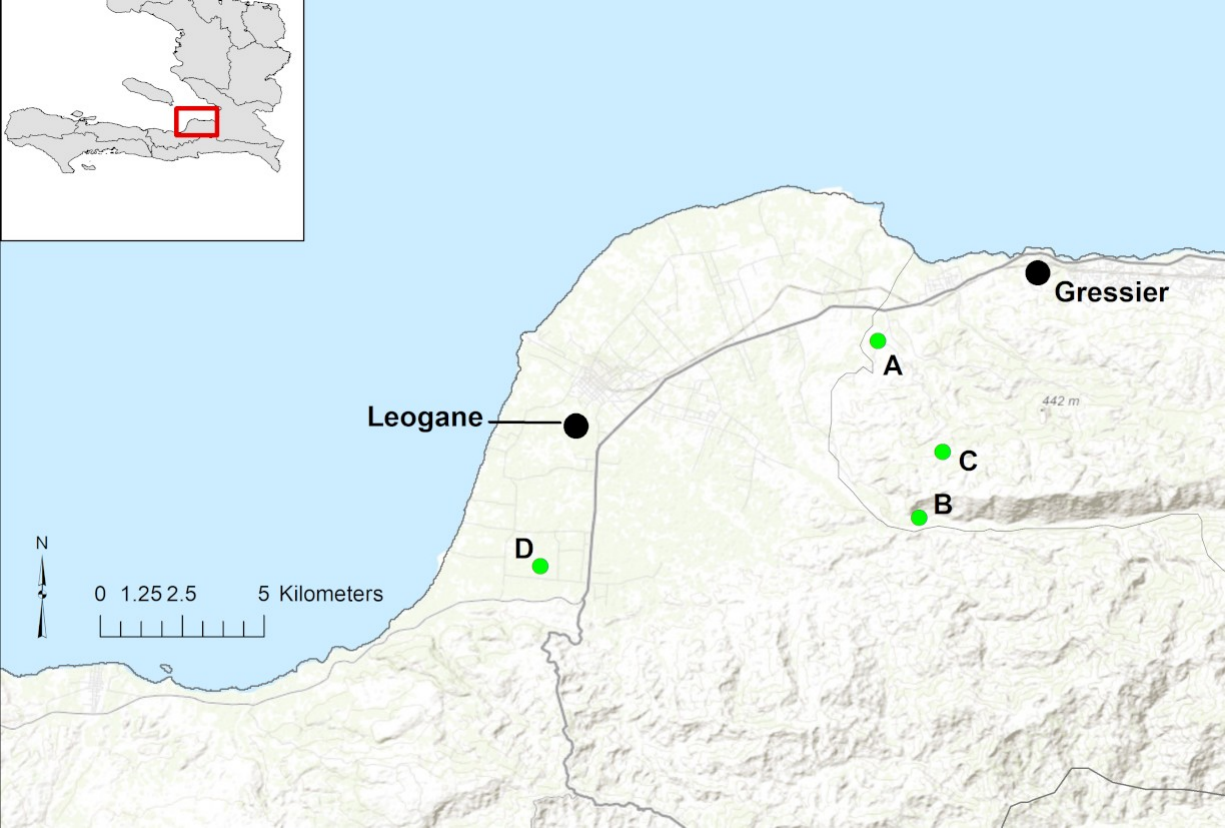
Map of schools enrolled in the study (green dots). Major towns in region are Leogane and Gressier (black dots) plotted over topography. National Route 2, the main highway through the area is identified with a bolded line and smaller roads appear lighter (gray). Base map data from ESRI Online, GIS Use Community and others.

Our group maintains regular surveillance for AFI through a school clinic serving a cohort of approximately 1,250 children who attend schools at the four campuses within the school system operated by the Christianville Foundation (a U.S.-based non-profit organization): this includes the main campus, housing grades K-13 (School A), and three small satellite elementary school campuses (Schools B, C, and D)(Fig 1). Clinic services are free, and serve as the primary source of medical care for students [7]. Since May, 2014, all school children seen in the clinic with AFI (defined as a subjective history of fever and/or fever on presentation in a child with no obvious source of infection) have been asked to provide a blood sample for viral screening [8–10]. All clinical data are collected and recorded by the clinic physician or nursing staff as part of routine clinical care, with data extracted from clinical charts for analysis.

### Ethics statement

The University of Florida IRB and the Haitian National IRB have approved all protocols, and written informed consent was obtained from parents or guardians of all study participants.

### Laboratory screening of samples

Whole blood was collected into yellow top (Acid Citrate Dextrose/ACD) tubes (Becton, Dickinson, and Company, Franklin Lakes, New Jersey), and an aliquot used to prepare blood smears for microscopic analyses for malaria parasites. To obtain plasma for virology analyses, a portion of the collected blood was centrifuged to pellet red and white blood cells, and the resulting plasma (ca. 650μL) transferred to sterile screw-top vials and stored at -80°C pending tests.

As there was a possibility that viruses such as yellow fever virus or EEEV might be present in samples, RNA extractions and virology work were performed in the Lednicky BSL3 laboratory at the University of Florida’s Emerging Pathogens Institute, Gainesville, FL. RT-PCR tests for the detection of chikungunya virus (CHIKV), dengue virus (DENV), and ZIKV-genomic RNAs (vRNAs) in the plasma were accomplished as previously described [10,11]. Briefly, vRNA was extracted from virions in the plasma using a QIAamp Viral RNA Mini Kit (Qiagen Inc., Valencia, CA), and the extracted vRNAs tested using previously described RT-PCR primers [12–14].

**Fig 2:**
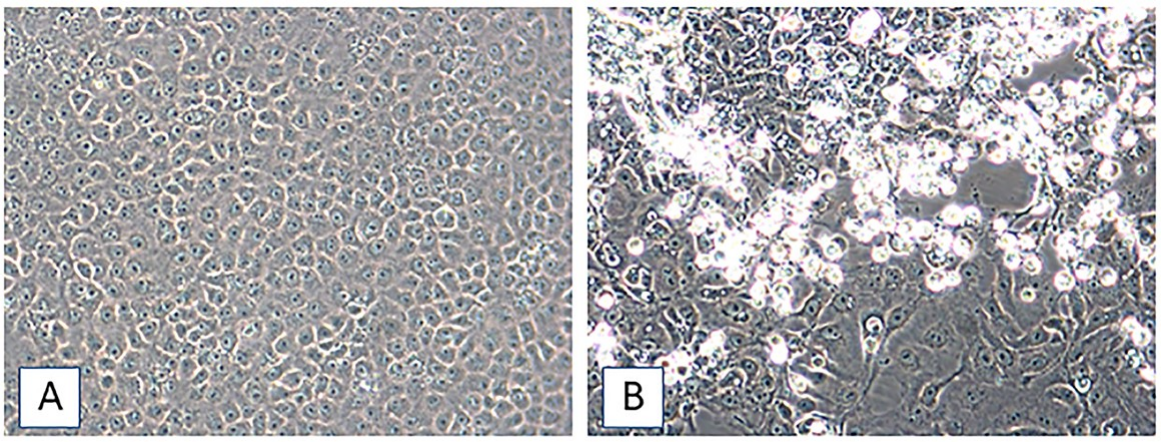
*Madariaga virus–*induced CPE in Vero E6 cells, 23 dpi. [A]. Mock-infected Vero E6 cells, 23 days post-seed. [B]. Vero E6 cells inoculated with plasma sample 1–1901. Infected cells develop dark, granulated cytoplasms with inclusion bodies, the cells enlarge, and dead cells detach from the growing surface. Images taken at an original magnification of 200X.

To explore the possibility that the aforementioned viruses were present at levels too low to detect by RT-PCR in samples negative for CHIKV, DENV, and ZIKV vRNAs, or that other viruses were the causative agents, aliquots of plasma were inoculated onto monolayers of LLC-MK2, MRC-5, and Vero E6 as previously outlined [10,11]. In samples from eight patients with AFI, batches of inoculated cells formed virus-induced cytopathic effects (CPE) within 6 to 22 days that were reminiscent of the CPE observed for alphaviruses such as CHIKV: the infected cells developed dark, granulated cytoplasms with inclusion bodies, became enlarged, then either detached from the growing surface or appeared to undergo apoptosis (Fig 2; virus strain list, and cell culture information, included as S1 Table). RT-PCR tests for the detection of CHIKV, DENV, and ZIKV were performed on vRNAs extracted from spent cell-media [10], and all were negative for the viruses. Therefore, they were next tested with universal primer systems for the detection and identification of both alpha- and flaviviruses [15]. A very weak alphavirus amplicon was generated, though the putative alphavirus amplicon did not correspond in size to the alphaviruses identified by de Morais Bronzoni *et al*. [6,15].

Suspecting MADV, primers used in our previous work were used to screen samples by RT-PCR, and specific amplicons formed by primer pairs were sequenced [6]. At the same time, aliquots of spent cell media from four cell cultures that displayed advanced CPE were treated with cyanase nuclease (RiboSolutions, Inc., Cedar Creek, Texas), and vRNA thereafter extracted from the treated material [10]. Synthesis of complementary DNA was achieved as previously described [9] using non-ribosomal hexamers to favor the reverse transcription of viral genomes over ribosomal RNA [16]. PCR was subsequently performed with random hexamers and One Taq DNA polymerase (New England Biolabs). Various prominent amplicons purified from a 2% agarose gel stained with ethidium bromide were sequenced. Both approaches revealed that the virus isolated was MADV.

Sanger sequencing was performed on vRNA from spent cell lysates from four patients to obtain the MADV consensus sequences using methods similar to a previously published genome walking approach using overlapping primers (S2 Table)[6,9,10]; GenBank numbers are MH359230, MH359231, MH359232, and MH359233 (S1 Table). Because primers described in our previous MADV article [6] were suboptimal, the primer list in S2 Table depicts primers that were purpose-designed for work with these strains.

### Phylogenetic analysis

All available MADV full genome sequences were downloaded from Genbank and codons aligned using MUSCLE [17]. Nucleotide substitution saturation and phylogenetic signal were assessed using DAMBE6 [18] and IQ-TREE [19] respectively (see S1 Fig). Maximum likelihood (ML) phylogeny was inferred using IQ-TREE based on the best-fitting model (GTR+F+G4) chosen according to Bayesian Information Criterion (BIC). Strong statistical support along the branches was defined as bootstrap > 90% based on 2,000 replicates of Ultrafast Bootstrap Approximation [20]. The ML tree was used to check for temporal signal with TempEst [21]. A time-scaled phylogeny for the MADV isolates was then inferred with BEAST [22] v.1.8.4. by using the HKY85 nucleotide substitution model [23], empirical base frequencies, and gamma distribution of site-specific rate heterogeneity. Strict and uncorrelated relaxed clocks, as well as constant size and Bayesian Skyline demographic priors were compared. The best-fitting model was chosen by calculating the Bayes Factor (BF) of marginal likelihood estimates (MLE) of different models, inferred with path sampling (PS) and stepping-stone sampling (SS) methods [22,24,25]. The strength of evidence against the null hypothesis (*H*_*0*_) in favor of the more complex model (*H*_*A*_)_,_ is evaluated according to the following guidelines: *ln*BF<2 no evidence; *ln*BF = 2–6—weak evidence; *ln*BF = 6–10—strong evidence, and *ln*BF>10 very strong evidence [25]. The best model for MADV isolates was strict molecular clock and Bayesian Skyline demographic prior (S3 Table). A Markov Chain Monte Carlo (MCMC) sampler was run for 200 million generations, sampling every 200,000, and proper mixing of the MCMC was confirmed when Effective Sampling Size (ESS) values for the parameter estimates were >200 using TRACER from the BEAST package. Maximum Clade Credibility (MCC) tree was extracted after 10% burn-in using Tree Annotator from the BEAST package.

## Results

From May, 2014, through June, 2016, 484 children seen at the Christianville School clinic were diagnosed as having AFI, and had a blood sample collected. From May, 2014, through February, 2015 (initial time period), 252 children with AFI were seen. As previously reported [8], confirmed laboratory diagnoses in this initial time period included CHIKV (31% of children), DENV1 (9%), DENV4 (13%), ZIKV (2%), and Mayaro virus (0.4%). No infections with MADV were identified.

From March, 2015, though June, 2016 (later time period), 232 children seen in the school clinic were diagnosed as having AFI and had plasma samples collected for analysis. In this later time period we identified eight AFI case patients who were infected with MADV. Cell cultures inoculated with plasma from the eight patients demonstrated typical alphavirus-induced CPE, as described above and shown in Fig 2. Spent culture media from the eight cultures displaying CPE were positive for MADV vRNA by RT-PCR, whereas mock-infected cells maintained in parallel were negative for MADV vRNA. Complete MADV consensus genome sequences were obtained when sequencing was performed on vRNA from spent cell lysates from four of the eight patients. Despite the very limited amounts of plasma available for the patients after the analyses described above, we were able to go back to the original samples and demonstrate positive RT-PCR signals for MADV in plasma from five of the eight patients; of these five patients, two had had fever when seen in the clinic, while three did not. We hypothesize that failure to identify RT-PCR signals in the other three samples (all of which were culture-positive for MADV) reflects very low quantities of the virus in the samples.

The first MADV infection occurred in an 8-year-old girl in April, 2015; she was one of seven children with AFI seen that month. The remaining seven cases were in 2016, including three in February (27% of 11 AFI cases that month); one in April (3% of 31 AFI cases); and 3 in May (4% of 75 AFI cases [May was the peak of the ZIKV epidemic in Haiti and at the school]). Six of the children were from school campus A, with one case from each of two of the other campuses. Mean age of case patients was 9 years (range 3–12 years); six were girls and two were boys.

When seen in the clinic, four children had temperatures above 37 degrees C; maximum recorded temperature in the clinic for a MADV case patient was 39 degrees C. The other four children were afebrile at the time of the clinic visit, but they or their parents reported subjective fever prior to coming to the clinic. Five reported cough, four reported headache, three reported nonspecific abdominal pain, and one reported myalgia; none reported arthralgias. When compared with previously reported symptom profiles of children with AFI in this cohort [8], symptoms most closely resembled those seen with laboratory-confirmed DENV infection. Arthralgias were significantly more common in patients with CHIKV infection than in those with MADV (64% in CHIKV vs. 0% in MADV, p<0001, Fishers Exact test, two tail).

One patient, who had a prior history of seizure activity, had an episode described as syncope followed by a short period of confusion and amnesia before being brought to the clinic. On exam, one child had a rash; one had conjunctivitis; and one was noted to have swollen tonsils. Physical exams were otherwise unremarkable. All children recovered without apparent sequelae, with six of the eight having remained in the school system through the time of preparation of this manuscript (2018), with continued follow-up by the clinic.

### Phylogenetic analysis

**Fig 3:**
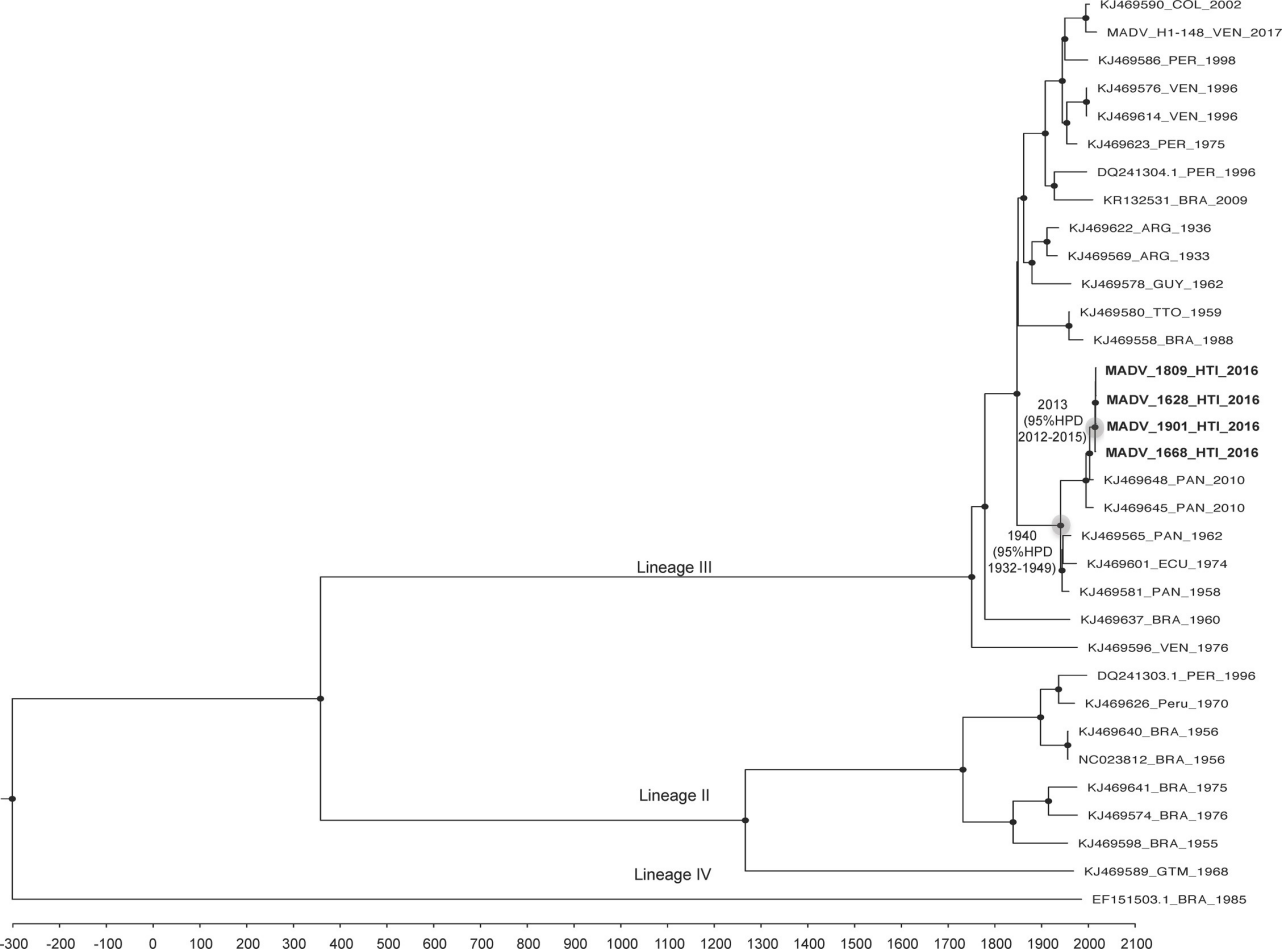
Bayesian phylogenetic tree based on the full genome of MADV isolates. The time-scaled MCC phylogeny was inferred based on a strict clock and a constant demographic prior model. MRCA for the Central American monophyletic sub lineage and the Haitian cluster are indicated with a light gray circle and tMRCA is reported at node. Robustness for internal branches defined as posterior probability PP> 0.9 is indicated by black circles at nodes.

The topology of the ML (S2 Fig) and MCC (Fig 3) phylogenies were in agreement. As noted previously, EEEV isolates cluster within four lineages: Lineage I constitutes the North American EEEV strains, while MADV fall into lineages II, III, and IV (Figs 3 and S2). The new isolates from Haiti cluster within Lineage III, which comprises isolates from Central and South America, and separate the Central American isolates from the Southern American ones, forming a Central American-Caribbean monophyletic sub lineage. The time of the most recent common ancestor (tMRCA) for the sub lineage was 1939, with a 95% High Posterior Density (HPD) interval of 1931–1948. Within this new sub lineage the new MADV isolates from Haiti cluster close to Panama isolates collected in 2010. The tMRCA for the MADV Haitian cluster was December 2013, with a 95% HPD interval of October 2012- January 2015, which corresponds to time window for the recent introduction of MADV in the island, likely from Panama. The evolutionary rate estimated for MADV was a 1.2 × 10^−4^ nucleotide substitution rate per year, in agreement with previous estimates calculated for this virus [1].

## Discussion

Reports of human infection with MADV are rare, although cross-sectional serologic studies (using plaque-reduction neutralization tests for confirmation) in Panama and Peru have reported seropositivity rates in human populations of between 2 and 5% [2,3,5], consistent with low-level MADV endemicity. Exposure may be substantially higher in epidemic settings, and/or with concurrent equine or animal epizootics: in recent work on seropositivity in household contacts of MADV and Venezuelan equine encephalitis virus cases during the MADV epidemic/epizootic in Panama in 2010, 19.4% of household contacts were seropositive for MADV [26]. In that same study, it was also noted that seroprevalence was comparable in all age groups, as might be expected if the virus had been recently introduced into Panama [26]. The phylogenetic analysis of our Haitian strains is consistent with recent introduction of MADV into Haiti, while our report of isolation of MADV from a child in Venezuela documents ongoing transmission in that country, concurrent with a possible equine epizootic [6]. Taken together, these observations are consistent with ongoing transmission/emergence of MADV at multiple sites in the Caribbean and South and Central America.

While most MADV case reports have involved patients with encephalitis, it is likely that the majority of infections occur in persons who are asymptomatic or who have only relatively mild disease [2,3,26]. This concept is supported by the previously noted work from our group in Venezuela [6], with identification of MADV (from a clade linked with Columbian and Venezuelan strains, distinct from our Haitian and Panamanian strains [Fig 3]) in a 12 year-old girl with headache and fever, rash, and conjunctivitis, but no evidence of encephalitis. In our Haitian study we saw a similar pattern of symptoms and signs, but with only one child with a rash, and only one with conjunctivitis. Carrera *et al*, in their study in Panama [3], used a pre-determined case definition for MADV of fever and headache. Only four of the eight patients in our study reported headache, suggesting that the Carrera case definition was overly restrictive. Interestingly, the pattern of symptoms in Haitian matched most closely with that previously reported from children in the same clinic with laboratory-confirmed DENV infections [8]; as with the DENV patients, the MADV patients were distinguished from the CHIKV patients by the lack of arthralgias. Overall, however, clinical presentation (in the absence of meningeal symptoms and signs) would appear to provide little assistance in diagnosing MADV infection.

*Culex (Melanoconion) pedroi* has been identified as a primary enzootic vector for MADV in the Amazon Basin [27]. The virus was also recovered from *Cx*. (Melanoconion) *taeniopus* in an epidemic outbreak in Panama [28], and in vector competence studies *Aedes fulvus* and *Psorophora albigenu* and *Ps*. *ferox* have been shown to be susceptible to and capable of transmitting the virus [29]. *Cx*. *pedroi* has not been previously identified in Haiti, but there are four known species in the Melanoconion subgenus including *Cx*. *atrutus*, *Cx*. *carcinophilus*, *Cx*. *erraticus*, and *Cx*. *pilosus* that are present [30], together with *Ps*. *ferox*, which is known to be a very aggressive biter of humans. It remains to be determined if these native Melanoconion subgenus mosquito species and/or *Ps*. *ferox* serve as vectors for MADV in Haiti. In an extensive survey by Vittor and colleagues [2] of possible reservoir hosts in Panama, evidence of infection was only found in rat species, with the highest seroprevalence in the short-tailed cane rat (*Zygodontomys brevivauda*; 8.7% seroprevalence, with one animal viremic for MADV) and the black rat (*Rattus rattus*; 3.9% seroprevalence). While Vittor found no evidence of infection in birds [2], there are suggestions in earlier studies that birds and reptiles can also be infected [1].

In Haiti, no prior data are available on MADV in vectors, animal reservoirs, or humans. While we cannot exclude the possibility that MADV was present in Haiti before the current case cluster, our phylogenetic studies are strongly suggestive of recent introduction of the virus into Haiti from Panama. Recent work by our group [8–11,31,32], and others, has underscored the apparent ease with which virus strains move among Caribbean and South and Central American countries. The drivers for this strain movement are varied. For Mayaro virus, we have shown a correlation between recent circulation of strains in this region and increased immigrant flow from Haiti to Peru and Brazil after the 2010 earthquake–and the counter-movement of peace-keeping troops from Brazil into Haiti during this same time period [32]. MADV is a little more complicated, as questions remain as to whether humans are a dead-end host, or if they can directly contribute to movement of the virus from one location to another. Over the past decade, there has been substantial movement of refugees from Haiti to and through Panama, as well as movement of Haitian workers to Panama; these population shifts may have provided an opportunity for movement of MADV from one country to the other. There is also the possibility that movement of strains was a function of movement of animal reservoirs (such as the black rat) on ships or in or on shipping containers; “hitch-hiking” of infected mosquitoes on airplanes is also a possibility [33]. At this point we know too little about the transmission and ecology of the virus to be able to predict its ability to move into other parts of the northern Caribbean, or areas such as Florida where North American EEEV is already endemic. Under these circumstances, the initiation of ongoing surveillance for MADV in humans, animals, and mosquitos throughout this region is clearly of public health importance.

## Supporting information

S1 Table *Madariaga virus*-positive plasma samples. (DOCX)

S2 Table Sequencing primers for MADV from Haiti. (DOCX)

S3 Table Molecular clock and demographic prior model testing with BEAST. (DOCX)

S1 Fig Substitution saturation and likelihood mapping in MADV sequence datasets. **(A)** Absence of substitution saturation indicated by plotting pairwise nucleotide transition (s) and transversion (v) substitutions against the Tamura and Nei 1993 (TN93) genetic distance **(B)** Likelihood mapping triangles indicating presence of phylogenetic signal by reporting over 30% of alternative topologies in the tips, as compared to unresolved quartets in the center, and partly resolved quartets in the edges.
(TIFF)

S2 Fig ML phylogenetic tree based on the full genome of MADV isolates. The ML tree was obtained based on the GTR+F+G4 nucleotide substitution model chosen as best-fit model according to BIC using IQ-TREE. Black circles at nodes indicate robust bootstrap support (BB> 90). Bar scale indicates genetic diversity.
(TIFF)
