# Supplementary material for: Emergence of Madariaga virus as a cause of acute febrile illness in children, Haiti, 2015-2016
Source: PLoS Negl Trop Dis. 2019 Jan 10;13(1):e0006972. doi: 10.1371/journal.pntd.0006972 (PMC6328082; doi:10.1371/journal.pntd.0006972)
Supplement: S1 Table — (DOCX) [file pntd.0006972.s001.docx]

**Supplemental Table S1:** ***Madariaga virus*-positive plasma samples.**

| **Plasma specimen #** | **Date Collected** | **Cell culture information** | | | | **GenBank accession # (complete virus genome sequence and host cell)** |
| --- | --- | --- | --- | --- | --- | --- |
|  |  | **LLCMK2** | **MRC-5** | **Vero E6** | **CPE (dpi)** |  |
| **1-1668** | 20-Apr-15 | **-** | **-** | **+** | 21 | MH359230 |
| **1-1802** | 05-Feb-16 | + | + | + | 20 |  |
| **1-1792** | 16-Feb-16 | - | + | + | 20 |  |
| **1-1809** | 19-Feb-16 | - | + | + | 6 | MH359231 |
| **1-1551** | 12-Apr-16 | - | + | + | 20 |  |
| **1-1628** | 23-May-16 | - | + | + | 21 | MH359232 |
| **1-1901** | 25-May-16 | + | + | + | 22 | MH359233 |
| **1-1903** | 25-May-16 | + | - | + | 20 |  |
| **Mock** | **N/A** | - | - | - | **-** |  |
| N/A; not applicable. | | | | | | |
