# Supplementary material for: Emergence of Madariaga virus as a cause of acute febrile illness in children, Haiti, 2015-2016
Source: PLoS Negl Trop Dis. 2019 Jan 10;13(1):e0006972. doi: 10.1371/journal.pntd.0006972 (PMC6328082; doi:10.1371/journal.pntd.0006972)
Supplement: S2 Table — (DOCX) [file pntd.0006972.s002.docx]

**Supplemental Table S2:** **sequencing primers for MADV from Haiti.**

| **Primer name** | **Primer sequence, 5’-3’** | **Nt in MADV ref. strain GB # KJ466958.1*** | **# nt mismatch with MADV ref. strain** |
| --- | --- | --- | --- |
| 5’ RACE roligo | rArGrCrArUrCrGrArGrUrCrGrGrCrCrUrUrGrU  rUrGrGrCrCrUrArCrUrGrG | N/A | NA |
| 5’RACE DNA primer | AGCATCGAGTCGGCCTTGTTGGCCTACTGG | N/A | NA |
| 5’RACE R | TTTGGAATGCGTGTGCCGGACAGGC | 270 - 246 | 0 |
| 1 F | ATAGGGTATGGTGTAGAGGCAG | 1 - 22 | 0 |
| 1 R | CTGCGCAATAATATTCTCTCCTC | 809 - 787 | 0 |
| 2 F | CCAACAAAGTCGTGTTCTCTGTC | 746 - 768 | 0 |
| 2 R | CTTCAACGGTTTCTCTCTCCAC | 1609 - 1588 | 1 |
| 3 F | CTGAAGCAGAAGCCATACGAGC | 1541 - 1562 | 0 |
| 3 R | GAACGCCTCGTCAATGTACAGTG | 2409 - 2387 | 2 |
| 4 F | GAACGGTGTCAAACATCCGG | 2361 - 2380 | 0 |
| 4 R | CTGGTGCACAGGAAGTTTAGGGC | 3209 - 3187 | 0 |
| 5F | GGGAAACCATTCCCGCTTTCAAG | 3137 - 3159 | 0 |
| 5R | GTGTGGAACCTTGATAGATG | 4009 - 3990 | 0 |
| 6 F | CCACCTACGCGATCAAGATCAAC | 3948 - 3970 | 1 |
| 6R | GGGATTGTATGCGGTGGTGAAGATGC | 4808 - 4783 | 2 |
| 7F | GTATTCGCTCTAAGTGCCCTGTCG | 4748 - 4771 | 0 |
| 7R | GAGTGTCGACGGATGAACTCG | 5609 - 5589 | 0 |
| 8F | GTACAGAAAGCCACCTGGCGTAG | 5538 - 5560 | 1 |
| 8R | CCTTTAGTTTAGTCACGTACTGC | 6418 - 6396 | 1 |
| 9 F | CTGGGAGACATTCAAGAACAACCC | 6345 - 6368 | 0 |
| 9R | CTCTTAAGGGGGTCTGCCACTC | 7217 - 7196 | 0 |
| 10F | GCGGAGGCTTCATCGTCGTGG | 7151 - 7171 | 0 |
| 10R | GGTCATAAATGCTTGCTTTCTTCAG | 8017 - 7993 | 0 |
| 11F | CTGCATGTAGAAGGAAGAATCGACAAT | 7946 - 7972 | 1 |
| 11R | GGGCACTGAGCAAGTAGGTAG | 88190 - 8799 | 0 |
| 12F | GCTCGCACGTCGGCACCGTGTTC | 8755 - 8778 | 1 |
| 12R | CACGTACACAGTCCCACGATAGTC | 9620 - 9597 | 1 |
| 13F | CCGCACGAGGTAGTAATTTATTACTAC | 9553 - 9579 | 0 |
| 13R | CATCACCGATCTTTGCTGGAGTTTCGC | 10417-10391 | 0 |
| 14F | GCGTCAGCTGGAGATCCGCTGATG | 10355-10378 | 0 |
| 14R | GCCCTAGTACTATAAATGCCGAAG | 11215-11192 | 0 |
| 15F | CATTCACGTCTGCGGTATCGGCCAC | 11132-11156 | 1 |
| 15R | GCCTTTTATAACACTATCGGCAGTGCATAATACTGCC | 11500 - 11464 | 1 |
| 3’ RACE F | gccacctgcgcagtgcataatg | 11340-11361 | 0 |
| T25A | TTTTTTTTTTTTTTTTTTTTTTTTTA | N/A | N/A |

*RERERENCE STRAIN: Madariaga virus strain MADV/Equus ferus caballus/PAN/MARV207963/1958, GenBank accession # KJ469581.1.
