# Supplementary material for: Emergence of Madariaga virus as a cause of acute febrile illness in children, Haiti, 2015-2016
Source: PLoS Negl Trop Dis. 2019 Jan 10;13(1):e0006972. doi: 10.1371/journal.pntd.0006972 (PMC6328082; doi:10.1371/journal.pntd.0006972)
Supplement: S3 Table — (DOCX) [file pntd.0006972.s003.docx]

**Supplemental Table S3: Molecular clock and demographic prior model testing with BEAST.**

| **Model** | **SS (MLE)** | ***ln*BF_SS_** | **PS (MLE)** | ***ln*BF_PS_** |
| --- | --- | --- | --- | --- |
| SC-CONST | -48891.95696 |  | -48892.15373 |  |
| RC-CONST | -49125.29822 |  | -49125.62714 |  |
| *SC-CONST vs*  *RC-CONST* |  | *-233.3412639* |  | *-233.4734159* |
| SC-BSP | -48884.5962 |  | -48884.98167 |  |
| RC-BSP |  |  |  |  |
| ***SC-CONST vs***  ***SC-BSP*** |  | ***7.360759006*** |  | ***7.172062304*** |
| *SC-BSP vs*  *RC-BSP* |  | *-236.6881327* |  | *-237.0846654* |

Best-fitting model indicated in bold. Model: molecular clock-demographic prior; SC= Strict clock; RC= Uncorrelated relaxed clock; CONST= Constant size demographic prior; SBP= Bayesian Skyline demographic prior; SS= Stepping Stone; MLE= marginal likelihood estimation; PS= Path Sampling; BF= Bayes Factor.
