# Supplementary figures and images for: Emergence of Madariaga virus as a cause of acute febrile illness in children, Haiti, 2015-2016
Source: PLoS Negl Trop Dis. 2019 Jan 10;13(1):e0006972. doi: 10.1371/journal.pntd.0006972 (PMC6328082; doi:10.1371/journal.pntd.0006972)

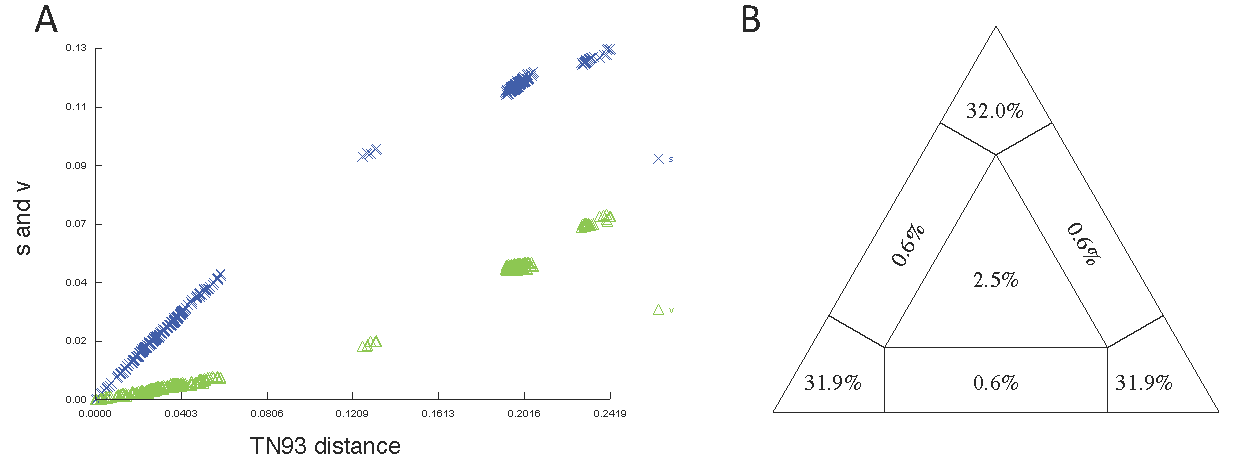

Supplement: S1 Fig — (A) Absence of substitution saturation indicated by plotting pairwise nucleotide transition (s) and transversion (v) substitutions against the Tamura and Nei 1993 (TN93) genetic distance (B) Likelihood mapping triangles indicating presence of phylogenetic signal by reporting over 30% of alternative topologies in the tips, as compared to unresolved quartets in the center, and partly resolved quartets in the edges. (TIFF) [file pntd.0006972.s004.tiff]

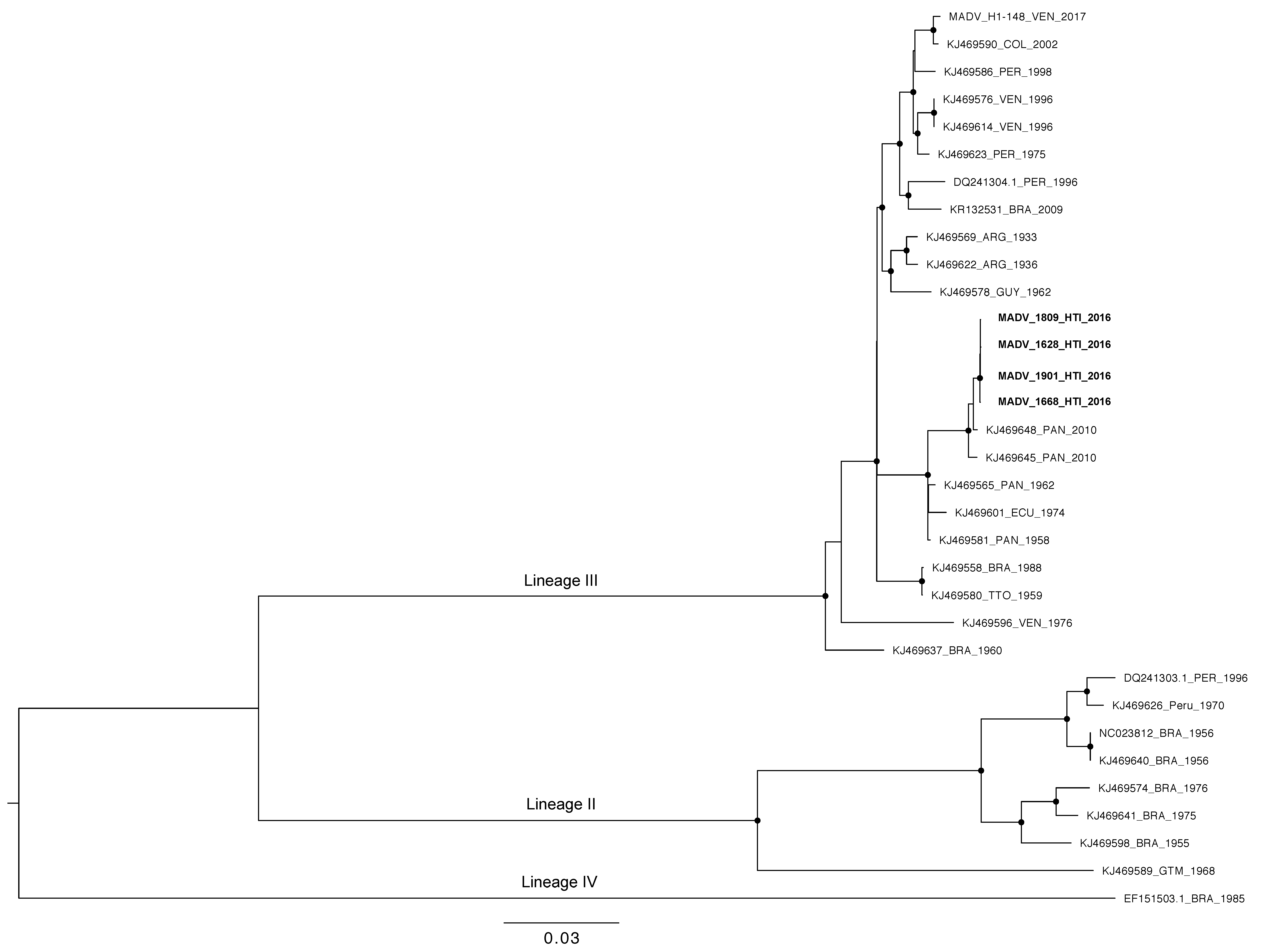

Supplement: S2 Fig — The ML tree was obtained based on the GTR+F+G4 nucleotide substitution model chosen as best-fit model according to BIC using IQ-TREE. Black circles at nodes indicate robust bootstrap support (BB> 90). Bar scale indicates genetic diversity. (TIFF) [file pntd.0006972.s005.tiff]
